# Adipose-Derived Mesenchymal Stem Cells Enhance Ovarian Cancer Growth and Metastasis by Increasing Thymosin Beta 4X-Linked Expression

**DOI:** 10.1155/2019/9037197

**Published:** 2019-10-20

**Authors:** Yijing Chu, Min You, Jingjing Zhang, Guoqiang Gao, Rendong Han, Wenqiang Luo, Tingting Liu, Jianxin Zuo, Fuling Wang

**Affiliations:** Department of Obstetrics and Gynaecology, The Affiliated Hospital of Qingdao University, Qingdao, China

## Abstract

As shown in our previous studies, growth and metastasis of ovarian cancer can be regulated by adipose-derived mesenchymal stem cells (ADSCs). However, the underlying mechanism has not yet been revealed. In this study, a proteomics analysis was performed to compare protein expression treated with and without ADSCs in ovarian cancer cells. Protein levels were altered in ovarian cancer cells due to the treatment of ADSCs. Thymosin beta 4 X-linked (TMSB4X) levels changed dramatically, and this protein was identified as one of the most important candidate molecules contributing to the tumour-promoting effects of ADSCs. Compared with the cells that are cultured in the normal growth medium, the TMSB4X levels cultured in ADSC-conditioned medium increased significantly in ovarian cancer cells. Furthermore, the growth and invasion of cancer cells were decreased, even in the ADSC-conditioned medium treatment group (*P* < 0.05), by the inhibition of TMSB4X. As shown in the bioluminescence images captured in vivo, increased ovarian cancer's growth and metastasis, along with elevated TMSB4X expression, were observed in the group of ADSC-conditioned medium, and the tumour-promoting effect of ADSCs was attenuated by the inhibition of TMSB4X. Based on our findings, increased TMSB4X expression may play a role in accelerating the ADSC-mediated proliferation, invasion, and migration of ovarian cancers.

## 1. Introduction

The epithelial ovarian cancer (EOC) is still the most deadly gynaecological tumour [1] because of late detection, local recurrence, and metastasis. Distant metastasis was diagnosed in approximately 61% of ovarian cancer patients, and the prognosis of EOC is poor [2]. Currently, the underlying mechanisms of omental metastasis in ovarian cancer are complex and ambiguous. Therefore, studies examining this topic are very important for the treatment of ovarian cancer.

The tumour microenvironment (TME) is composed of mesenchymal stromal cells, immune cells, and noncellular components around the tumour tissue [3]. Recently, the TME has been reported to have significant contributions to the metastasis of ovarian cancer [4, 5]. In our previous study, by using ADSCs in the omentum, the growth and invasion of ovarian cancer were increased significantly, indicating that the ovarian cancer progression is promoted [6]. Besides, the prometastatic TME in the omentum that promotes omental metastasis is formed during this process; however, the underlying mechanism is not entirely understood.

Proteomics analysis is a promising method for identifying various proteins related to the regulation of cancer. The isobaric tags for relative and absolute quantitation- (iTRAQ-) based proteomic analysis is a powerful tool [7]. In this study, TMSB4X is identified as a differentially expressed protein in ovarian cancer cells after ADSC treatment. The study is aimed at investigating the role of TMSB4X when ADSCs promote ovarian cancer's growth and the underlying mechanisms involved in the study.

## 2. Methods and Materials

### 2.1. Preparation of Conditioned Medium and Cell Culture

When ADSCs isolated from the omentum reached 80% confluence, we replaced the medium with DMEM/F12 (Gibco, Carlsbad, CA) lacking FBS and cultured the cells for an additional 24 h. After centrifugation at 1,200 × g for 12 min and filtering through a 0.22-micron filter (Millipore, Billerica, MA), the medium was collected for subsequent experiments.

Human omental tissues were removed from donors who were treated for a benign gynaecological disease using abdominal surgery and did not present with other diseases [6]. All ADSCs were used at passages 3-5 in the present study. Every procedure was carried out by the ethical guidelines of the Affiliated Hospital of Qingdao University, China. ES2, HO8910, and SKOV3 cells, which were obtained from the Type Culture Collection China Centre, were cultured and used for experiments. Cells were cultured in a 37°C incubator with a 5% CO_2_ atmosphere. The DMEM/F12 containing 10% FBS was used to culture all three ovarian cancer cell lines.

### 2.2. Identification of ADSCs

The expression of cellular markers, including a hematopoietic marker (CD34) and mesenchymal markers (CD105, CD73, and CD90), in ADSCs (passage 3) was examined using flow cytometry (CD90-FITC, CD73-APC, CD105-PECy7, and CD34-PE antibodies from eBioscience were used, San Diego, CA).

Moreover, the ability of ADSCs in differentiating into osteoblasts and adipocytes was assessed. ADSCs cultured in 6-well plates grew to approximately 50% confluence. Then, the ADSCs were cultured in osteogenic differentiation medium or adipogenic differentiation medium (Gibco, Carlsbad, CA) for 21 days. ADSCs were stained with alizarin red S and oil red O to verify the osteoblast and adipocyte differentiation of ADSCs, respectively.

### 2.3. iTRAQ-Based Proteomic Analysis

We homogenized and sonicated the ovarian cancer cells in 0.5% sodium dodecyl sulfate (SDS). After centrifugation at 20,000 × g for 30 min, a Pierce BCA protein assay kit (Sigma-Aldrich, St. Louis, MO, USA) is used to determine the protein concentration. The FASP method was used to digest and label proteins, as described previously [8]. Then, the labelled peptides were collected, dried by vacuum centrifugation, and then fractionated with an Agilent 1260 infinity II HPLC Purifier system (GE Healthcare). Four millilitres of buffer A (10 mM HCOONH4 and 5% ACN, pH 10.0) was used to reconstitute the peptide mixtures. The reconstituted peptide mixtures were eluted with a gradient from 100% buffer A to 100% buffer B (10 mM HCOONH4 and 85% ACN, pH 10.0) at a 1 mL/min flow rate for 90 min. Besides, after collection, the UV absorbance of each fraction was monitored at 214 nm, and fractions were concentrated by vacuum centrifugation. 60 *μ*L of 0.1% (*v*/*v*) trifluoroacetic acid was used to reconstitute the concentrated fractions.

An automated Easy nLC and a Q-Exactive™ Plus mass spectrometer (both from Thermo Fisher Scientific, Inc., MA, USA) were used to perform liquid chromatography-tandem mass spectrometry (LC-MS/MS). In 0.1% formic acid, the peptide mixture was reconstituted and loaded onto the C18-reverse phase column (Thermo Fisher Scientific, Inc., 50 *μ*m inner diameter, 15 cm long, nanoviper). Then, at a 300 nL/min flow rate with a linear gradient of buffer B (0.1% formic acid and 80% acetonitrile), the mixture was separated for 60 minutes. Next, a full mass spectrum with a scan range of 350–1,800 (*m*/*z*) was recorded and complete peptides with a resolution of 70,000 were obtained in the Orbitrap mass analyser. Using high-energy collision dissociation, ten of the strongest precursor ions with a mass resolution of 17,500 at *m*/*z* 2 were separated for MS/MS fragmentation. We used a 0.1% under fill ratio and 30 eV normalized collision energy. The target value for Automatic Gain Control was 3*e*6. The maximum ion injection times (IT) of the MS/MS scan and the full scan were 50 ms and 45 ms.

Proteome Discoverer version 2.1 software (Thermo Fisher Scientific, Inc.) Mascot (version 2.6.0) was used to perform the LC-MS/MS analysis. The raw MS data were used for protein identification.

### 2.4. shRNA Transfection

Three ovarian cancer cell lines were cultured for one day in 10 cm plates until they reached a subconfluent status. Then, using Lipofectamine 2000 (Invitrogen, Carlsbad, CA), we transfected the cancer cells with a TMSB4X-specific shRNA and a nontargeting scrambled shRNA (shNC) (Hanbio, Shanghai, China). An shRNA sequence targeting human TMSB4X (target sequence: 5′-CCGGGAAGACAGAGACGCAAGAGAACTCGAGTTCTCTTGCGTCTCTGTCTTCTTTTTTG-3′) or a nontargeting control (target sequence: 5′-GCACTACCAGAGCTAACTCAGATAGTACT-3′) was used. The transfected cells were isolated as single clones after puromycin treatment to establish lines with stable TMSB4X knockdown.

### 2.5. Quantitative Real-Time PCR (qRT-PCR)

TMSB4X shRNA-transfected SKOV3, HO8910, and ES2 cells were treated with ADSC-conditioned medium (CM) for 24 h. Subsequently, we extracted the total RNA from transfected cancer cells using a Trizol reagent (Takara, Japan). Then, we used a reverse transcription kit (Invitrogen) to synthesize the complementary DNAs. Quantitative real-time PCR (qRT-PCR) was performed by using SYBR Premix Ex Taq (Takara, Japan) and an ABI 7500 Sequencing Detection System. GAPDH served as a control. The primer sequences were used as follows: TMSB4X—5′-ACAAACCCGATATGGCTGAG-3′ (forward) and 5′-GAAGGCAATGCTTGTGGA-3′ (reverse); GAPDH—5′-ATGGGGAAGGTGAAGGTCG-3′ (forward) and 5′-GGGGTCATTGATGGCAACAATA-3′ (reverse). Three separate reactions were performed for each marker.

### 2.6. Scratch-Wound Assay

Three lines of ovarian cancer cells were cultured to 100% confluence. In the confluent monolayer with a 200 *μ*L pipette tip, linear scratch wounds were created. The phosphate-buffered saline (PBS) was used to gently wash the cells. The serum-free medium, which blocks cancer cell growth, was used to replace the medium. Cells were cultured for 12 h or 24 h to allow them to migrate into the wound. Images were captured under a microscope at the starting time point of 0 h, 12 h, and 24 h to compare the healed areas.

### 2.7. Ovarian Cancer Cell Invasion Assay

In 24-well plates (Corning, NY, USA), three lines of ovarian cancer cells (5 × 105) were seeded in Transwell chambers placed. The 8 *μ*m pore membranes in the 24-well plates were covered with 50 *μ*L of a 1 : 10 dilution of Matrigel™ matrix. Cancer cells were cultured on the membranes for 6 h. Two types of medium were infused into the lower chamber: ADSC CM supplemented with 10% FBS or normal culture medium supplemented with 10% FBS (control). Transwell assays were performed while culturing ovarian cancer cells without FBS for one day. Next, 4% paraformaldehyde was used to fix all cancer cells. Cancer cells that penetrated through the membranes were analysed. The number of cells that migrated through the membrane in each high magnification field was calculated. Every experiment was performed for three times.

### 2.8. Western Blotting

Ovarian cancer cells were lysed on ice for 12 min by using RIPA buffer (Sigma-Aldrich, St. Louis, MO, USA), while centrifuging the cell lysate at 12,000 × g and treating it with LDS sample buffer. Then, protein mixtures were separated on SDS-PAGE gels and transferred from the gel to the polyvinylidene fluoride (PVDF) membrane (Bio-Rad, Hercules, CA). Next, the membrane was blocked with 5% skim milk.

Subsequently, the blocked membrane was incubated with the primary rabbit monoclonal antibody against human TMSB4X (1 : 1,000 dilution; Cell Signaling Technology, USA) or GAPDH (1 : 1,000 dilution; Proteintech, Chicago, IL). Then, the membrane was incubated with secondary antibodies (1 : 1,000; CST, Danvers, MA). We detected and quantified the protein-antibody complexes using a chemiluminescence detection system (Bio-Rad, Hercules, CA).

### 2.9. Cell Proliferation Analysis

A density of 5,000 cells/well was in the 96-well plates, in which three lines of ovarian cancer cells were cultured. The CCK-8 reagent (Thermo Fisher Scientific, Inc., MA, USA) was used to measure ovarian cancer cell proliferation daily. We added the CCK-8 reagent to each well, and the cancer cells were cultured for an additional 1.5 hours. Then, colorimetric assays were performed by measuring the absorbance (OD value) of each well at a wavelength of 450 nm in a microplate reader. The growth curves were determined in three independent experiments.

### 2.10. Orthotopic Ovarian Cancer Model

By using an in vivo imaging system, an orthotopic mouse model of ovarian cancer was established and the tumour cell behaviour was monitored. Briefly, the ovarian cancer cell line of firefly luciferase expression (SKOV3-Luc) was generated through transfecting the SKOV3 cell line with lentiviral particles (GenePharma Co., Shanghai, China) and selecting cells with high luciferase expression using puromycin. The SKOV3-Luc cell was harvested and then maintained in PBS (10,000 cells/*μ*L) on ice. We then anaesthetized and performed surgery on the animals as described and performed in a previously released video [9]. An incision was made in the left side of the midline above the mouse's ovary. Under a microscope, the ovarian fat pad was removed and the ovary was fixed to insert a needle into the bursa. 5 *μ*L of the cell suspension was injected between the cyst and ovary by gently pushing the plunger of the syringe. We then gently replaced the reproductive tract and sutured the skin. Recovering animals were monitored and provided a safe heat source. The thirty female BALB/c-nu nude mice that underwent this surgery were randomly divided into the following 5 groups: control+PBS, shTMSB4X+CM, shTMSB4X+PBS, shNC+CM, and shNC+PBS. ADSC CM or PBS was injected intraperitoneally on day 7 after surgery.

The behaviours of the SKOV3 cells were monitored using Caliper Life Sciences instruments. Before being anaesthetized with isoflurane, animals were intraperitoneally injected with a solution of the firefly luciferase substrate D-luciferin. All mice underwent imaging studies weekly after surgery for 5 weeks. The bioluminescence intensity in growing tumours was analysed by using the Living Image software (Lumina II Living Image 4.3). In order to sum the counts for a whole tumour area, the total counts were normalized to the image acquisition time (photon/second).

### 2.11. Statistical Analysis

Statistical analyses were performed using one-way analysis of variance or two-tailed Student's *t*-test, and all data are reported as the means ± standard deviations from at least three independent experiments. A significant difference was indicated by considering a *P* value < 0.05.

## 3. Results

### 3.1. The Growth, Migration, and Invasion of Ovarian Cancer Cells Promoted by ADSCs

**Figure 1:**
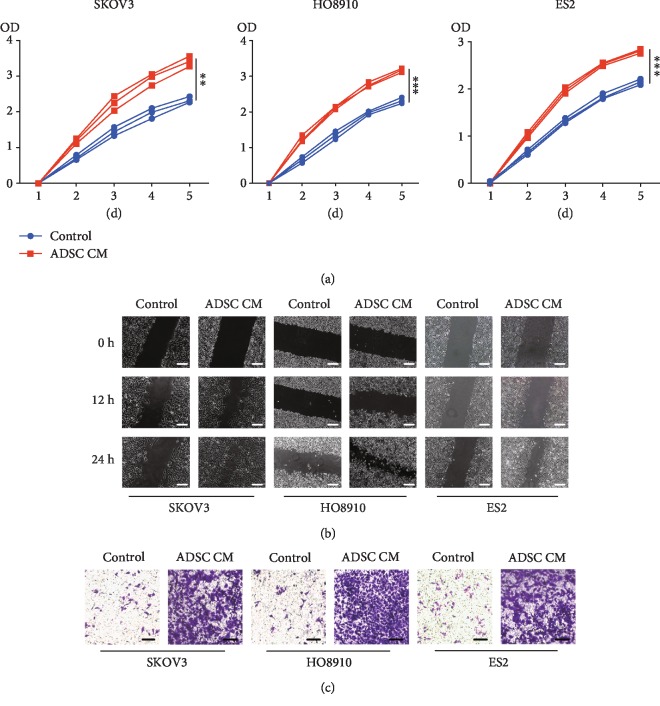
ADSCs promote the proliferation, migration, and invasion of ovarian cancer cells in vitro. (a) Representative results of CCK-8 assays in SKOV3, HO8910, and ES2 cells are shown. Cancer cells were treated with ADSC CM. (b) Representative results of scratch-wound assays for the detection of ovarian cancer cell mobility (scale bar, 100 *μ*m). (c) Representative results of Transwell assays in ovarian cancer cells are shown. The number of cancer cells that migrated through the 8 *μ*m Transwell membrane pores was counted to determine changes in the invasive capabilities in response to ADSC CM (scale bar, 50 *μ*m).

First, we isolated ADSCs and identified the multidirectional differentiation ability and surface markers of ADSCs ([Supplementary-material supplementary-material-1]). Next, the ovarian cancer cells' proliferation, migration, and invasion, which were treated with or without ADSC CM, were examined by using CCK-8, scratch-wound, and Transwell assays, respectively, to assess the effects of ADSCs on ovarian cancer (Figures 1(a)–1(c)). The ADSC CM-treated ovarian cancer cells exhibited significantly increased proliferation compared with cancer cells (all *P* < 0.001). Based on the results of the scratch-wound assays, ADSC CM increased the number of migrating cancer cells after 24 h; the migrated SKOV3 number, HO8910 cells, and ES2 cells treated with CM increased by 53.0%, 67.5%, and 54.2%, respectively, compared with the number of migrated cells cultured with normal medium (*P* < 0.001). Similarly, the invaded SKOV3 number, HO8910 cells, and ES2 cells increased approximately 7-fold after ADSC CM treatment. Thus, ADSCs promoted the ovarian cancer cells' proliferation, invasion, and migration.

### 3.2. The Proteomic Profile of the Ovarian Cancer Cells Regulated by ADSCs

We used iTRAQ to assess changes in protein levels induced by ADSC CM. The levels of 149 proteins were altered (fold changes ≥ 1.5 or ≤0.67) ([Supplementary-material supplementary-material-1]). Among these proteins, 75 upregulated and 84 downregulated proteins were detected in ADSC CM-treated ovarian cancer cells, respectively. The top 10 upregulated proteins included thymosin beta 4 X-linked (TMSB4X), thymosin beta-10, and forkhead-associated domain-containing protein 1 (Table 1). Of these ten proteins, the most significant change was observed in the level of TMSB4X, with a 2.7-fold increase in ADSC CM-treated ovarian cancer cells. Thus, we studied the role of TMSB4X in the effect of ADSCs on promoting ovarian cancer growth.

### 3.3. TMSB4X Was Overexpressed in Ovarian Cancer Cells Cultured with ADSC CM

**Figure 2:**
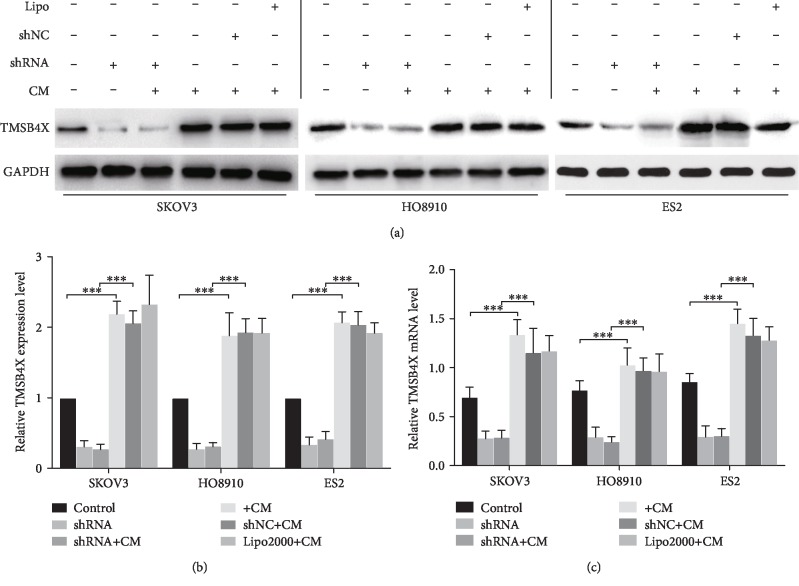
ADSC increases the expression of TMSB4X in ovarian cancer cells, and the expression levels of TMSB4X in shRNA-transfected ovarian cancer cells are decreased. (a) Cancer cells were transfected with TMSB4X shRNA or shNC. TMSB4X expression was detected in three lines of transfected ovarian cancer cells cultured alone or with ADSC CM by western blot. (b) The histograms show the quantification of the relative expression of TMSB4X. (c) Significant increases in TMSB4X mRNA were found in SKOV3, HO8910, and ES2 cells treated with ADSC CM by qRT-PCR. The results are expressed as the means ± SD.

First, we confirmed that TMSB4X was overexpressed in primary and metastatic ovarian cancer using immunohistochemistry ([Supplementary-material supplementary-material-1] and [Supplementary-material supplementary-material-1]). Then, in ovarian cancer cells, the effects of ADSCs on TMSB4X expression were investigated. The qRT-PCR was used to assess the mRNA levels, and western blotting was used to evaluate the levels of the TMSB4X protein in the ADSC CM-treated ovarian cancer cells. Compared with cells cultured with normal medium, TMSB4X was expressed at much higher levels in the ovarian cancer cells treated with ADSC CM (Figure 2(a), all *P* < 0.001). Similarly, the presence of ADSC CM significantly increased the levels of theTMSB4X protein and mRNA in ovarian cancer cells compared with the untreated group (Figure 2(c), all *P* < 0.001). Based on these data, ADSCs induced ovarian cancer cells to overexpress TMSB4X.

### 3.4. ADSCs Increased Ovarian Cancer Cell Proliferation by Increasing TMSB4X Expression

**Figure 3:**
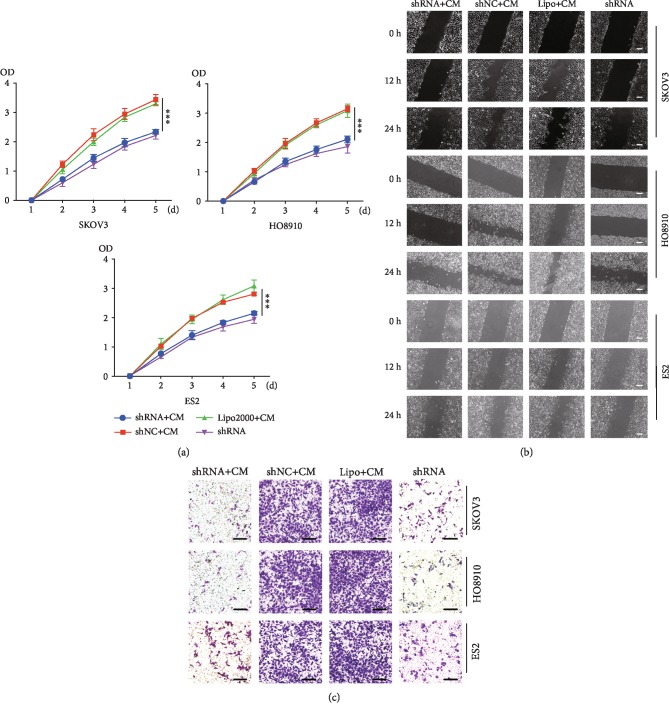
ADSCs enhance the proliferation, migration, and invasion of ovarian cancer cells by increasing TMSB4X expression in vitro. (a) Cell proliferation was evaluated by a CCK-8 assay. Three ovarian cancer cell lines transfected with TMSB4X shRNA were treated with ADSC CM. Ovarian cancer cells transfected with shNC/lipo2000 and treated with ADSC CM served as the positive control, and cancer cells transfected with TMSB4X shRNA and cultured in normal growth medium served as the negative control. (b) Representative results of scratch-wound assays for ovarian cancer cell migration abilities. (c) Cancer cell invasion abilities were determined by Transwell assay (scale bar, 50 *μ*m). ^∗∗∗^*P* < 0.001.

After confirming that ADSCs induced TMSB4X expression in ovarian cancer cells, we subsequently studied the underlying mechanism. We inhibited TMSB4X expression with a specific shRNA to determine TMSB4X's role in the tumour-promoting effects of ADSCs. The western blot and qRT-PCR results confirmed that the shRNA inhibited the TMSB4X expression in the three ovarian cancer cell lines (see Figures 2(a)–2(c)). Then, ADSC CM was used to treat the transfected ovarian cancer cell, and the growth was assessed using a CCK-8 assay. Even in the presence of ADSC CM, the TMSB4X inhibition suppressed the growth of cancer cells (Figure 3(a)).

Next, we examined the TMSB4X's role in ovarian cancer cell migration and invasion after treatment with or without ADSC CM. The migration and invasion of ovarian cancer cells were significantly increased by the treatment with ADSC CM, and these increases were reduced in shRNA-transfected cancer cells (Figures 3(b) and 3(c)). Taken together, ADSCs may accelerate ovarian cancer cell's proliferation, migration, and invasion by increasing TMSB4X expression.

### 3.5. ADSCs Promoted Ovarian Cancer Growth and Metastasis In Vivo by Increasing TMSB4X Expression

**Figure 4:**
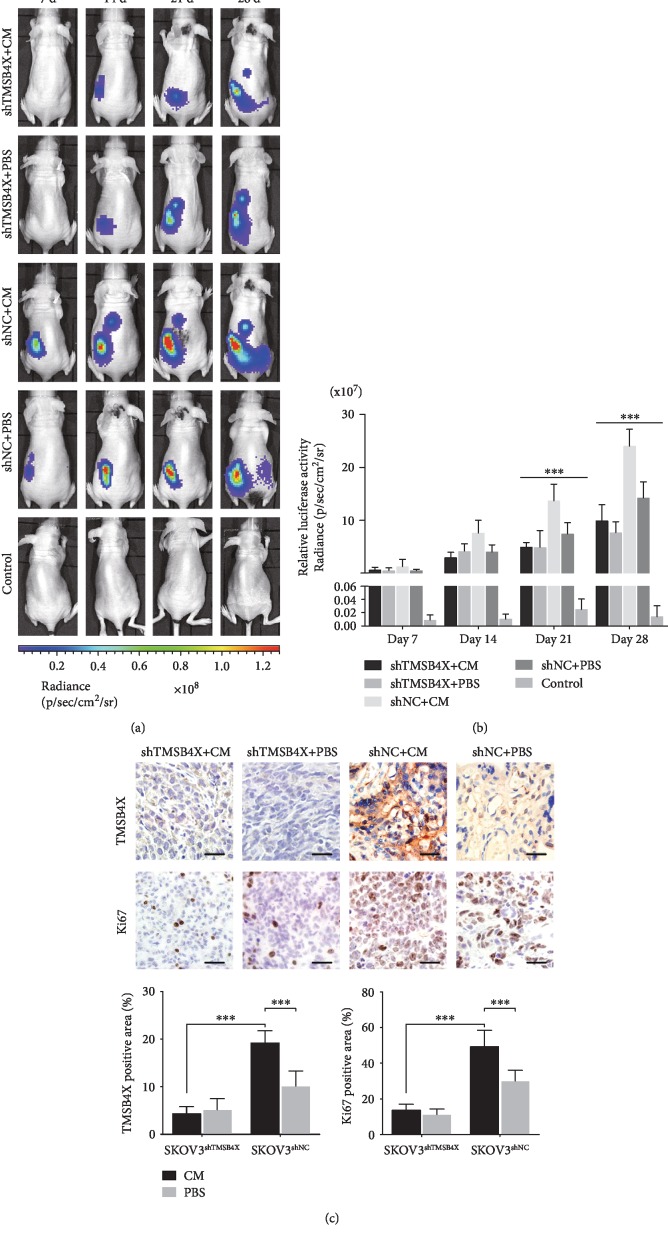
ADSCs enhance the proliferation, migration, and invasion of ovarian cancer cells by increasing TMSB4X expression in nude mouse models. (a) An in vivo imaging system was used to monitor SKOV3 xenograft luminescence activity, which represented tumour growth and metastasis. (b) Living Image software was used to analyse tumour bioluminescence intensity weekly. Quantitative results regarding the normalized image counts are shown. (c) Immunohistochemical analysis results regarding Ki67 and TMSB4X expression in the orthotopic tumour xenografts are shown. Quantitative results of Ki67 and TMSB4X expression-positive area in xenografts on the right panel. ^∗^*P* < 0.05, ^∗∗^*P* < 0.01, and ^∗∗∗^*P* < 0.001.

We performed a set of xenograft experiments to examine the role of TMSB4X in the effects of ADSCs on promoting ovarian cancer growth in vivo. We verified that the orthotopic models were successfully established seven days after surgery using an in vivo imaging system. Much stronger bioluminescent signals were detected in the shNC+CM group than in the other groups starting at week 2, and the tumour signals differ dramatically among the four groups (Figures 4(a) and 4(b), *P* < 0.001). Compared to the other groups, the ability of ADSCs to promote tumour growth was inhibited significantly in the shTMSB4X group. In addition, metastasis to the contralateral ovary, liver, and spleen was more frequently observed in the shNC+CM group than in the other groups (Figure 4(a)).

We examined Ki67 expression in xenograft tumour tissues to analyse the proliferation of transplanted ovarian cancer cells. Ki67 was expressed at high levels in ovarian tumour tissues from the shNC+CM group. Higher levels of TMSB4X were detected in the shNC+CM group than in the other groups (Figure 4(c)). Thus, ADSCs promoted ovarian cancer's growth and metastasis and a blockade of TMSB4X expression reversed this effect.

## 4. Discussion

The metastatic sites of malignant tumours are particular, and premetastatic niches are now being recognized as playing a prominent role in metastatic progression in ectopic organs. In our previous studies, it was shown that ADSCs promote the proliferation and invasion of ovarian cancer cells and play a role in the formation of omental premetastatic niches in ovarian cancer [6]. Increasing evidence supports that ADSCs regulate many changes in the TME during tumour progression by secreting soluble factors and extracellular vesicles [10–14]. However, we know little about the exact mechanisms by which ADSCs promote ovarian cancer cells' growth and metastasis. TMSB4X, an actin-binding protein, displays numerous functions in many physiological and pathological processes [15, 16]. TMSB4X proteins regulate intracellular signal transduction and has been proved to overexpress in various cancers, including colorectal, lung, gastric, pancreatic, and squamous cell cancers [17–21]. It plays as a hypoxic reaction regulator to control cancer cell migration and is associated with anti-inflammation and antiapoptosis [15, 22, 23]. A protein expression analysis in ovarian cancer cell lines treated with or without ADSC CM showed that compared to the cancer cells cultured with normal medium, TMSB4X is expressed at approximately 2.7-fold higher levels in the ADSC CM group. Our findings from this study are consistent with previous reports. Next, the effects of ADSCs on promoting ovarian cancer growth were investigated and the role of TMSB4X was determined in this process.

Consistent with the findings from previous studies, the proliferation, migration, and invasion of the ovarian cancer cell treated with ADSC CM were significantly increased compared to cancer cells cultured with the normal medium in the present study. Additionally, compared with normal culture medium, ADSC CM substantially increased TMSB4X expression in ovarian cancer cells. Moreover, in vitro and in vivo, the effect of ADSCs on promoting tumour proliferation, migration, and invasion was suppressed by TMSB4X knockdown in ovarian cancer cells. Based on our results, the ovarian cancer-promoting effects of ADSCs may be at least partially attributed to the expression of TMSB4X in ovarian cancer cells. Our findings provide a novel insight into the mechanism by which ADSCs promote the metastasis of ovarian cancer.

ADSCs can promote tumour growth and drive metastatic progression [24]. The communication between ADSCs and other cells was facilitated through the molecules secreted by ADSCs. This communication participates in the pathological processes of breast and ovarian cancer [25–28]. In our unpublished research, we examined the culture supernatant of ADSCs using a Multiplex analysis, and IL-1/6 were present at extremely high levels, and the STAT3 signalling pathway was activated in cancer cells, which were treated with ADSC CM. TMSB4X expression in ovarian cancer cells may be regulated with the ADSC-secreted IL-1/6, but more studies are required to confirm these findings. The epithelial-mesenchymal transition (EMT) mediated by TMSB4X facilitates cancer cell motility [29, 30]. Previously, TMSB4X was reported to induce the EMT by activating integrin-linked kinase (ILK) and the TGF beta signalling pathway to promote tumour progression [31]. However, the available evidence did not reveal a role for TMSB4X in regulating the interaction between the TME and primary tumour. In the present study, TMSB4X expression was regulated by ADSCs, forming a TME that is favourable for ovarian cancer metastasis.

In conclusion, ADSCs promote the proliferation, migration, and invasion of ovarian cancer cells at least partly by increasing TMSB4X expression in ovarian cancer cells. TMSB4X is a candidate regulator of cancer progression and metastasis, such as ADSC-mediated tumour progression that is involved in regulating the interaction between the TME and primary tumour. Our findings provide insights that may assist with the development of cancer-specific targeted preventive strategies in the future. Identifying the mechanisms by which ADSCs increase TMSB4X expression in ovarian cancer cells needs further studies.

## Figures and Tables

**Table 1:** Top 10 increased expressed protein in ovarian cancer cells treated with ADSC-conditioned medium.

Accession	Description	Gene name	Ratio _(ADSC/NC)_	*P* value
A2VCK8	Thymosin beta 4 X-linked	TMSB4X	2.70	0.00142
P63313	Thymosin beta-10	TMSB10	2.68	0.00012
B1AJZ9	Forkhead-associated domain-containing protein 1	FHAD1	2.56	0.00071
B4DRB1	cDNA FLJ50735, highly similar to calsyntenin-3		2.53	0.00338
Q9BY12	S phase cyclin A-associated protein in the endoplasmic reticulum	SCAPER	2.48	0.00085
Q8IY33	MICAL-like protein 2	MICALL2	2.45	0.00080
Q9Y4F3	Meiosis regulator and mRNA stability factor 1	MARF1	2.37	0.00044
Q9UK12	Zinc finger protein 222	ZNF222	2.28	0.00085
J3QRZ1	Nuclear distribution protein nudE-like 1	NDEL1	2.26	0.01320
B2R6V9	cDNA, FLJ93141, highly similar to Homo sapiens coagulation factor XIII, A1 polypeptide (F13A1), mRNA		2.17	0.00033

## Data Availability

The data used to support the findings of this study are included within the article and available from the corresponding author upon request.

## Supplementary Materials

Supplementary 1 Figure S1: characterization of primary ADSCs derived from human omentum and TMSB4X expression in ovarian cancer tissues.

Supplementary 2 Table S1: list of the differentially expressed proteins of SKOV3 cells induced by ADSC-conditioned medium.

Supplementary 3 Table S2: patient population for experiments.

## References

[1] Webb P. M., Jordan S. J. (2017). Epidemiology of epithelial ovarian cancer. *Best Practice & Research Clinical Obstetrics & Gynaecology*.

[2] Richardson D. L. (2019). New and novel therapies for gynecologic cancers. *Seminars in Oncology Nursing*.

[3] Roma-Rodrigues C., Mendes R., Baptista P., Fernandes A. (2019). Targeting tumor microenvironment for cancer therapy. *International Journal of Molecular Sciences*.

[4] Turley E. A., Wood D. K., McCarthy J. B. (2016). Carcinoma cell hyaluronan as a “portable” cancerized prometastatic microenvironment. *Cancer Research*.

[5] Wu S., Zheng Q., Xing X. (2018). Matrix stiffness-upregulated LOXL2 promotes fibronectin production, MMP9 and CXCL12 expression and BMDCs recruitment to assist pre-metastatic niche formation. *Journal of Experimental & Clinical Cancer Research*.

[6] Chu Y., Tang H., Guo Y. (2015). Adipose-derived mesenchymal stem cells promote cell proliferation and invasion of epithelial ovarian cancer. *Experimental Cell Research*.

[7] Aggarwal S., Yadav A. K. (2016). Dissecting the iTRAQ data analysis. *Statistical Analysis in Proteomics*.

[8] Wisniewski J. R., Zougman A., Nagaraj N., Mann M. (2009). Universal sample preparation method for proteome analysis. *Nature Methods*.

[9] Cordero A. B., Kwon Y., Hua X., Godwin A. K. (2010). In vivo imaging and therapeutic treatments in an orthotopic mouse model of ovarian cancer. *Journal of Visualized Experiments*.

[10] Danan D., Lehman C. E., Mendez R. E. (2018). Effect of adipose-derived stem cells on head and neck squamous cell carcinoma. *Otolaryngology–Head and Neck Surgery*.

[11] Baasse A., Juerss D., Reape E., Manda K., Hildebrandt G. (2018). Promoting effects of adipose-derived stem cells on breast cancer cells are reversed by radiation therapy. *Cytotechnology*.

[12] Wang Y., Chu Y., Yue B. (2017). Adipose-derived mesenchymal stem cells promote osteosarcoma proliferation and metastasis by activating the STAT3 pathway. *Oncotarget*.

[13] Wang Y., Liu J., Jiang Q. (2017). Human adipose-derived mesenchymal stem cell-secreted CXCL1 and CXCL8 facilitate breast tumor growth by promoting angiogenesis. *Stem Cells*.

[14] Koellensperger E., Bonnert L. C., Zoernig I. (2017). The impact of human adipose tissue-derived stem cells on breast cancer cells: implications for cell-assisted lipotransfers in breast reconstruction. *Stem Cell Research & Therapy*.

[15] Kuzan A. (2016). Thymosin *β* as an actin-binding protein with a variety of functions. *Advances in Clinical and Experimental Medicine*.

[16] Goldstein A. L., Hannappel E., Sosne G., Kleinman H. K. (2012). Thymosin *β*_4_: a multi-functional regenerative peptide. Basic properties and clinical applications. *Expert Opinion on Biological Therapy*.

[17] Huang D., Wang S., Wang A., Chen X., Zhang H. (2016). Thymosin beta 4 silencing suppresses proliferation and invasion of non-small cell lung cancer cells by repressing Notch1 activation. *Acta Biochimica et Biophysica Sinica*.

[18] Ryu Y. K., Lee Y. S., Lee G. H., Song K. S., Kim Y. S., Moon E. Y. (2012). Regulation of glycogen synthase kinase-3 by thymosin beta-4 is associated with gastric cancer cell migration. *International Journal of Cancer*.

[19] Zhang Y., Feurino L. W., Zhai Q. (2008). Thymosin beta 4 is overexpressed in human pancreatic cancer cells and stimulates proinflammatory cytokine secretion and JNK activation. *Cancer Biology & Therapy*.

[20] Chi L. H., Chang W. M., Chang Y. C. (2017). Global proteomics-based identification and validation of thymosin Beta-4 X-linked as a prognostic marker for head and neck squamous cell carcinoma. *Scientific Reports*.

[21] Lee S. Y., Park M. J., Lee H. K. (2017). Increased expression of thymosin *β*_4_ is independently correlated with hypoxia inducible factor-1*α* (HIF-1*α*) and worse clinical outcome in human colorectal cancer. *Journal of Pathology and Translational Medicine*.

[22] Gupta S., Kumar S., Sopko N., Qin Y., Wei C., Kim I. K. (2012). Thymosin *β*4 and cardiac protection: implication in inflammation and fibrosis. *Annals of the New York Academy of Sciences*.

[23] Hong Y., Yao Q., Zheng L. (2017). Thymosin *β*4 attenuates liver fibrosis via suppressing Notch signaling. *Biochemical and Biophysical Research Communications*.

[24] Ridge S. M., Sullivan F. J., Glynn S. A. (2017). Mesenchymal stem cells: key players in cancer progression. *Molecular Cancer*.

[25] de Lope L. R., Alcíbar O. L., Amor López A., Hergueta-Redondo M., Peinado H. (2018). Tumour–adipose tissue crosstalk: fuelling tumour metastasis by extracellular vesicles. *Philosophical Transactions of the Royal Society B: Biological Sciences*.

[26] Gilbert C. A., Slingerland J. M. (2013). Cytokines, obesity, and cancer: new insights on mechanisms linking obesity to cancer risk and progression. *Annual Review of Medicine*.

[27] Cho J. A., Park H., Lim E. H. (2011). Exosomes from ovarian cancer cells induce adipose tissue-derived mesenchymal stem cells to acquire the physical and functional characteristics of tumor-supporting myofibroblasts. *Gynecologic Oncology*.

[28] Lin R., Wang S., Zhao R. C. (2013). Exosomes from human adipose-derived mesenchymal stem cells promote migration through Wnt signaling pathway in a breast cancer cell model. *Molecular and Cellular Biochemistry*.

[29] Fu X., Cui P., Chen F. (2015). Thymosin *β*4 promotes hepatoblastoma metastasis via the induction of epithelial-mesenchymal transition. *Molecular Medicine Reports*.

[30] Hong K. O., Lee J. I., Hong S. P., Hong S. D. (2016). Thymosin *β*4 induces proliferation, invasion, and epithelial-to-mesenchymal transition of oral squamous cell carcinoma. *Amino Acids*.

[31] Morita T., Hayashi K.'i. (2018). Tumor progression is mediated by thymosin-*β*4 through a TGF*β*/MRTF signaling axis. *Molecular Cancer Research*.

