# Supplementary figures and images for: Adipose-Derived Mesenchymal Stem Cells Enhance Ovarian Cancer Growth and Metastasis by Increasing Thymosin Beta 4X-Linked Expression
Source: Stem Cells Int. 2019 Oct 20;2019:9037197. doi: 10.1155/2019/9037197 (PMC6855023; doi:10.1155/2019/9037197)

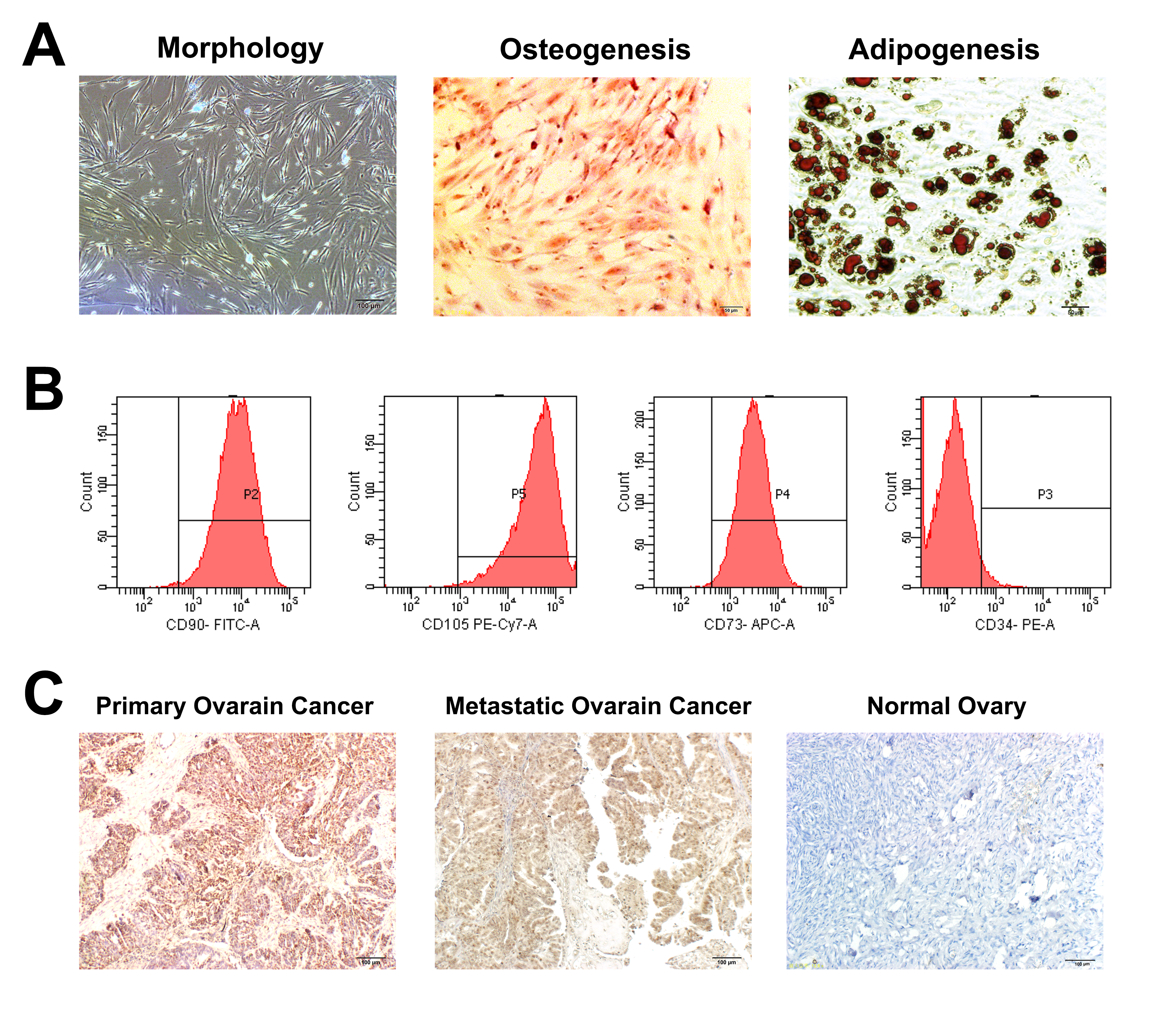

Supplement: Supplementary 1 — Figure S1: characterization of primary ADSCs derived from human omentum and TMSB4X expression in ovarian cancer tissues. [file 9037197.f1.tif]
