# Supplementary material for: Adipose-Derived Mesenchymal Stem Cells Enhance Ovarian Cancer Growth and Metastasis by Increasing Thymosin Beta 4X-Linked Expression
Source: Stem Cells Int. 2019 Oct 20;2019:9037197. doi: 10.1155/2019/9037197 (PMC6855023; doi:10.1155/2019/9037197)
Supplement: Supplementary 3 — Table S2: patient population for experiments. [file 9037197.f3.docx]

Table S2

Patient population for experiments

| Type of tissue | Tissue condition | Number of patients | Number of positive patients |
| --- | --- | --- | --- |
| Normal ovary | Paraffin | 4 | 0 |
| Primary ovarian cancer | Paraffin | 20 | 18 |
| Metastatic ovarian cancer | Paraffin | 18 | 18 |
